# Supplementary figures and images for: Emergence and retention of a collective memory in cockroaches
Source: PLoS One. 2023 Jul 6;18(7):e0287845. doi: 10.1371/journal.pone.0287845 (PMC10325095; doi:10.1371/journal.pone.0287845)

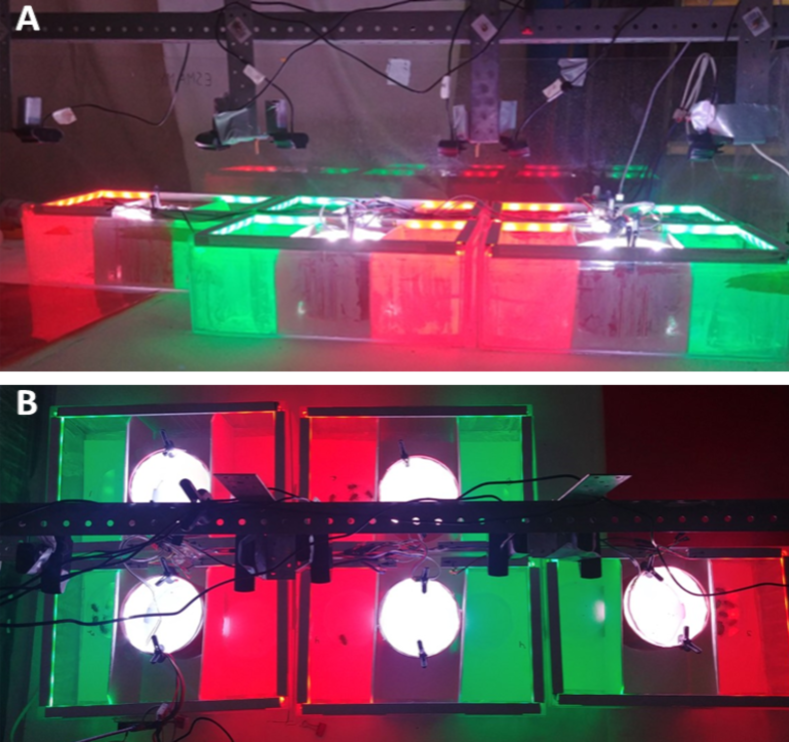

Supplement: S1 Fig — (TIF) [file pone.0287845.s001.tif]

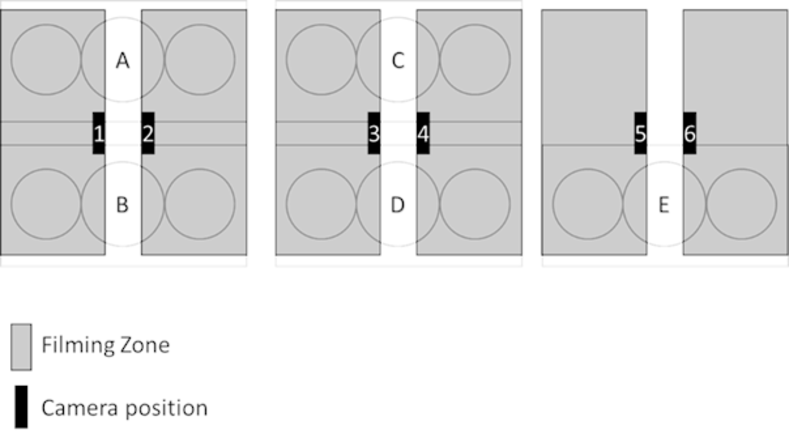

Supplement: S2 Fig — (TIF) [file pone.0287845.s002.tif]

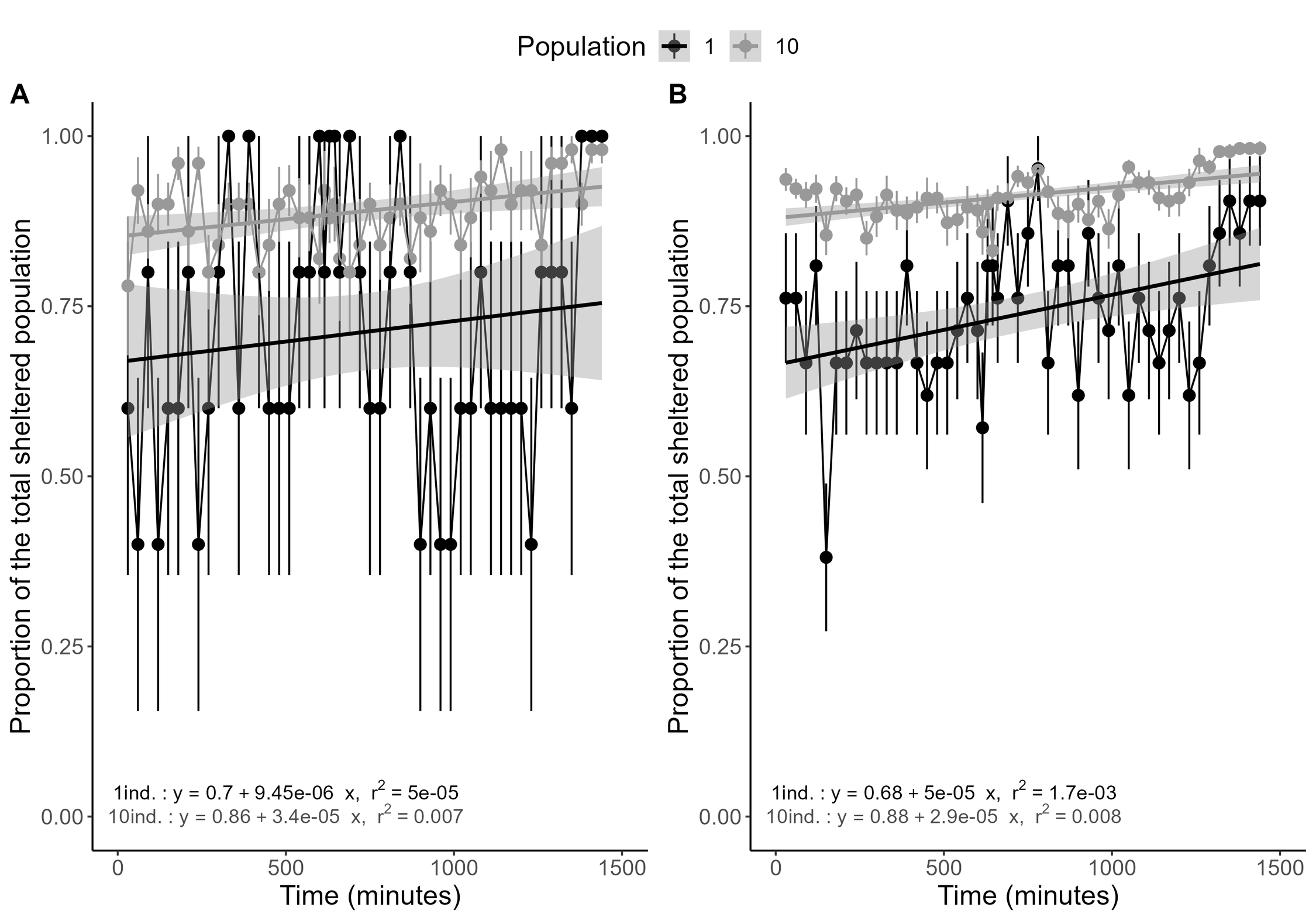

Supplement: S3 Fig — Mean ± SEM of isolated individuals (black) and of groups (grey). (TIF) [file pone.0287845.s003.tif]

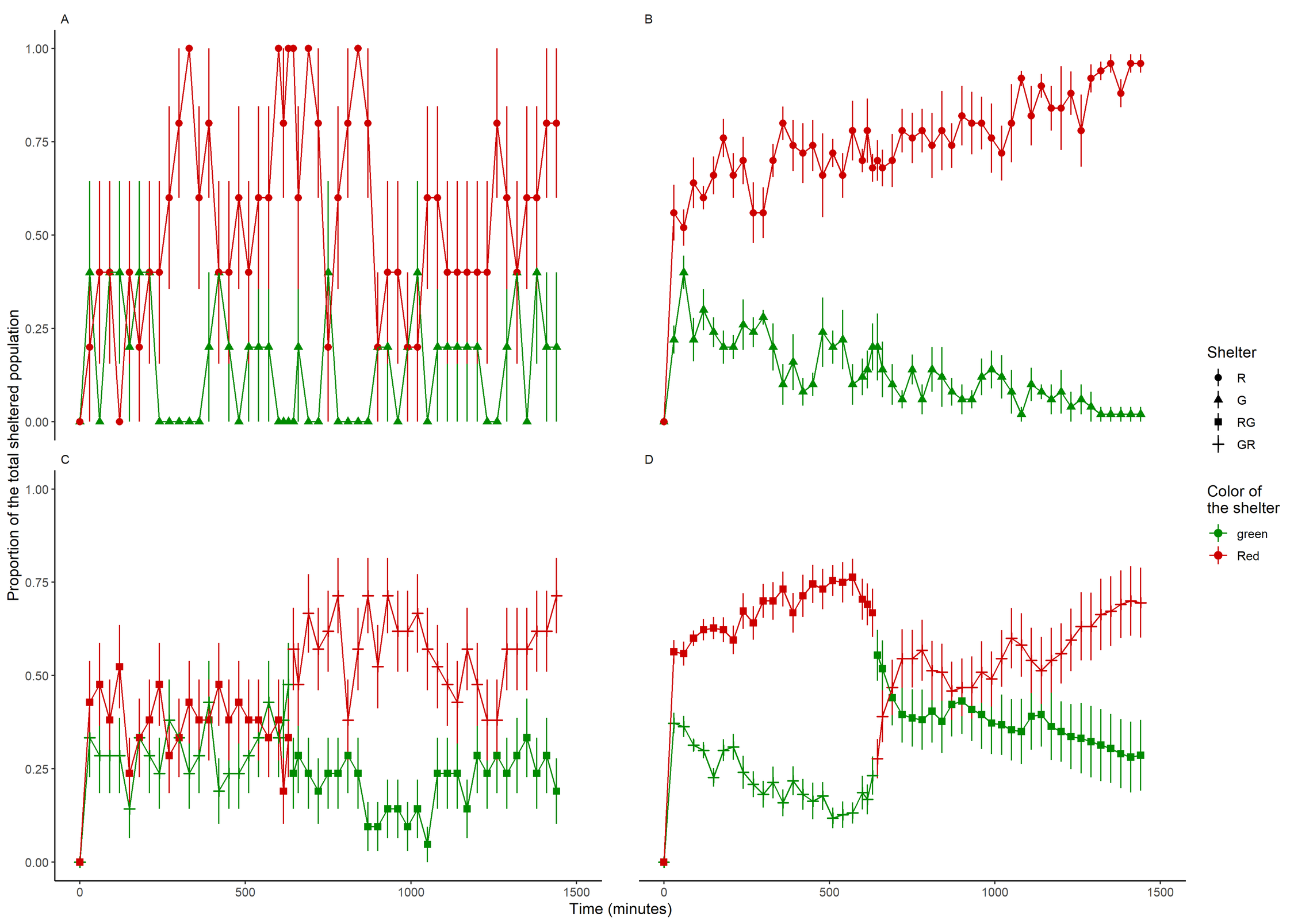

Supplement: S4 Fig — (TIF) [file pone.0287845.s004.tif]

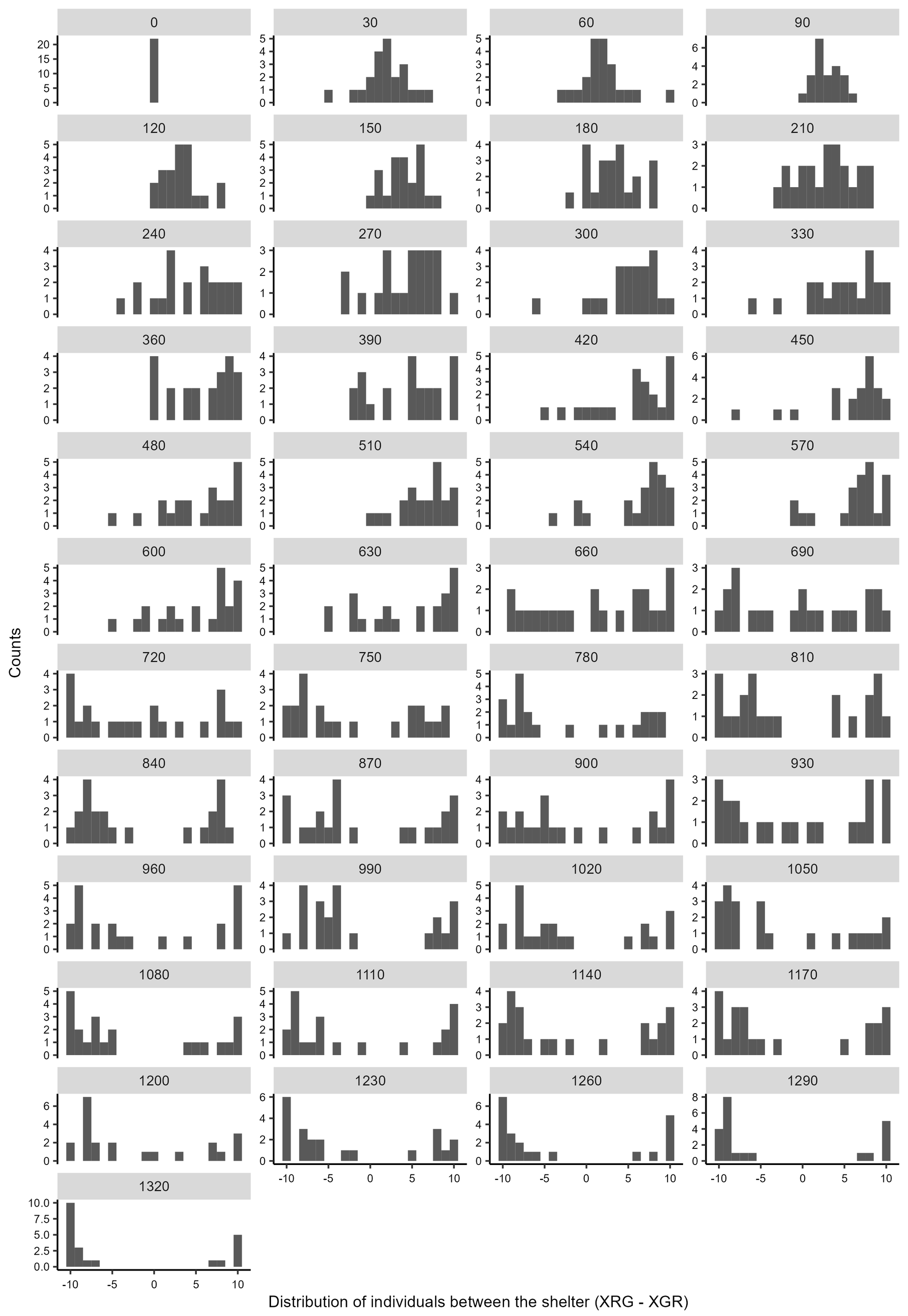

Supplement: S5 Fig — (TIF) [file pone.0287845.s005.tif]

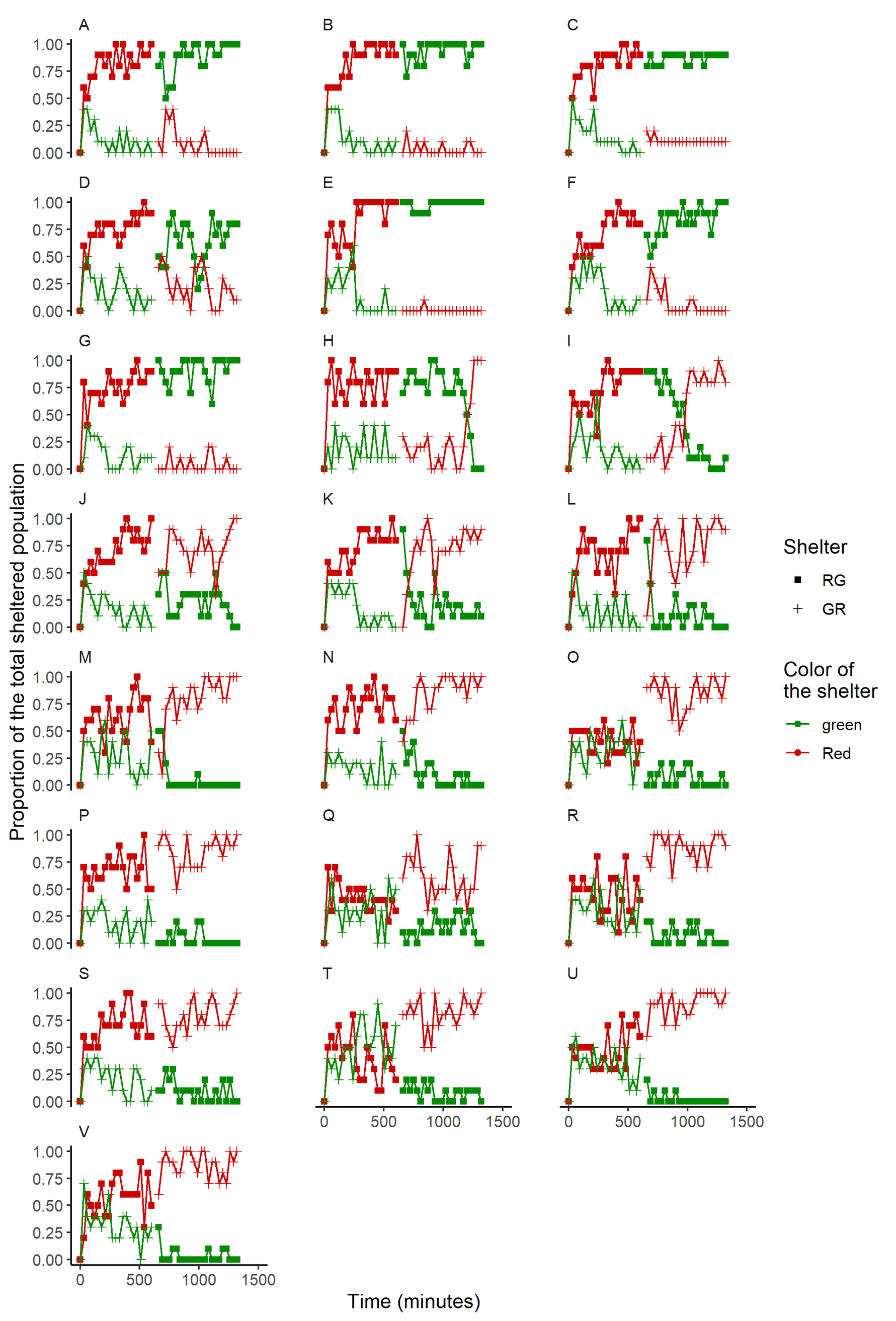

Supplement: S6 Fig — A-G) Consensual groups in the RG at 600 and at 1320 minutes. (TIF) [file pone.0287845.s006.tif]

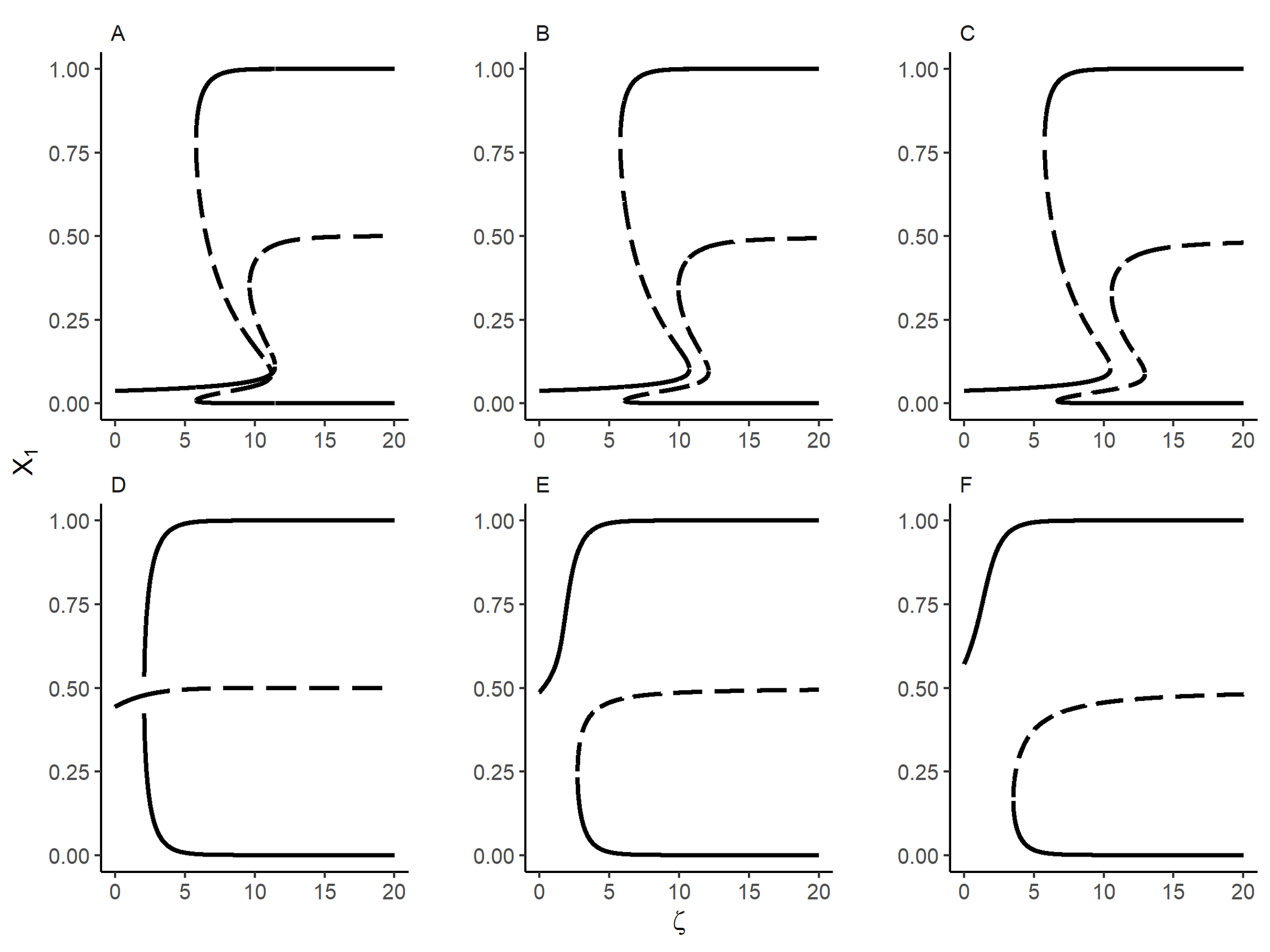

Supplement: S7 Fig — (TIF) [file pone.0287845.s007.tif]

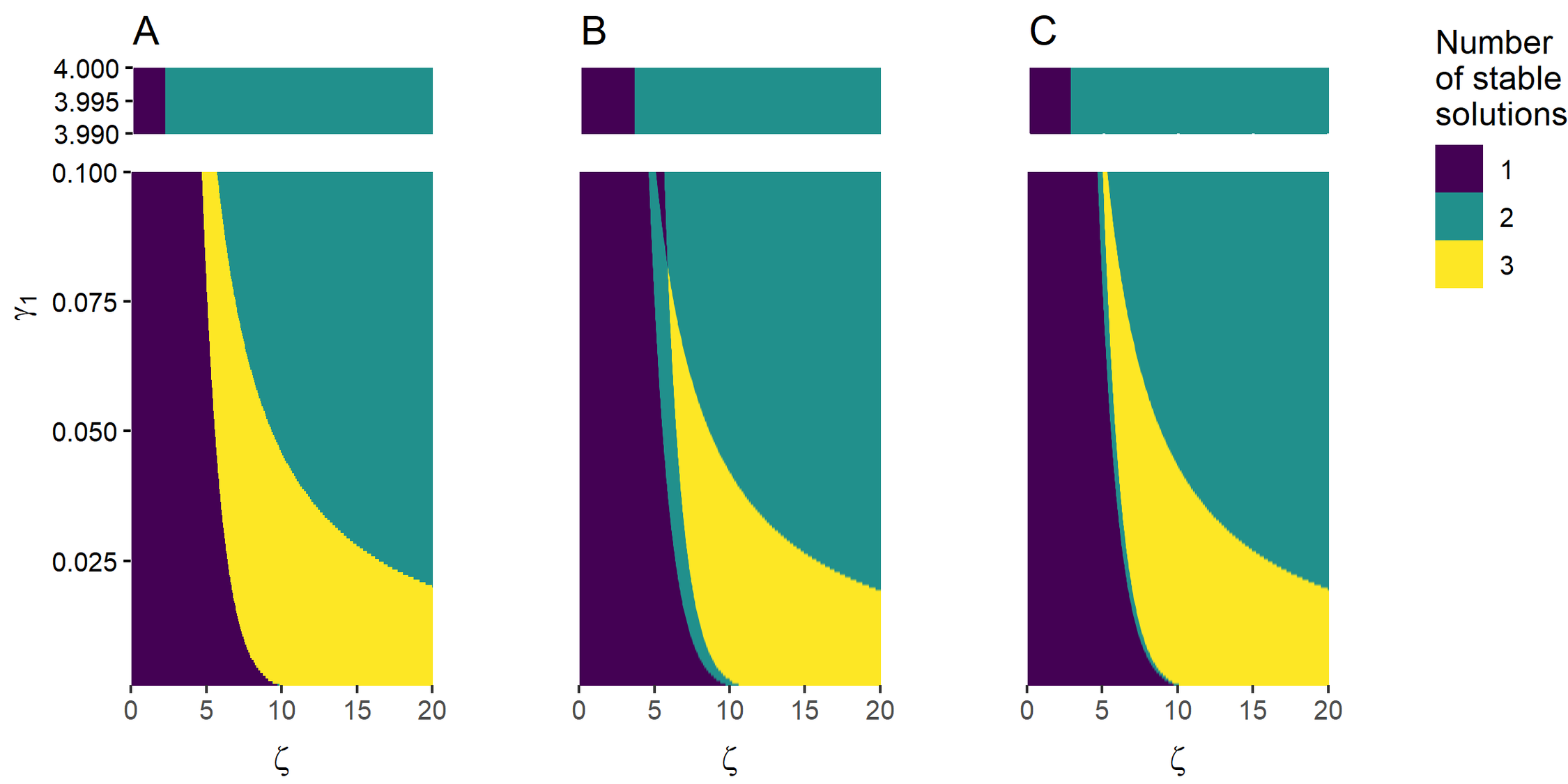

Supplement: S8 Fig — (TIF) [file pone.0287845.s008.tif]

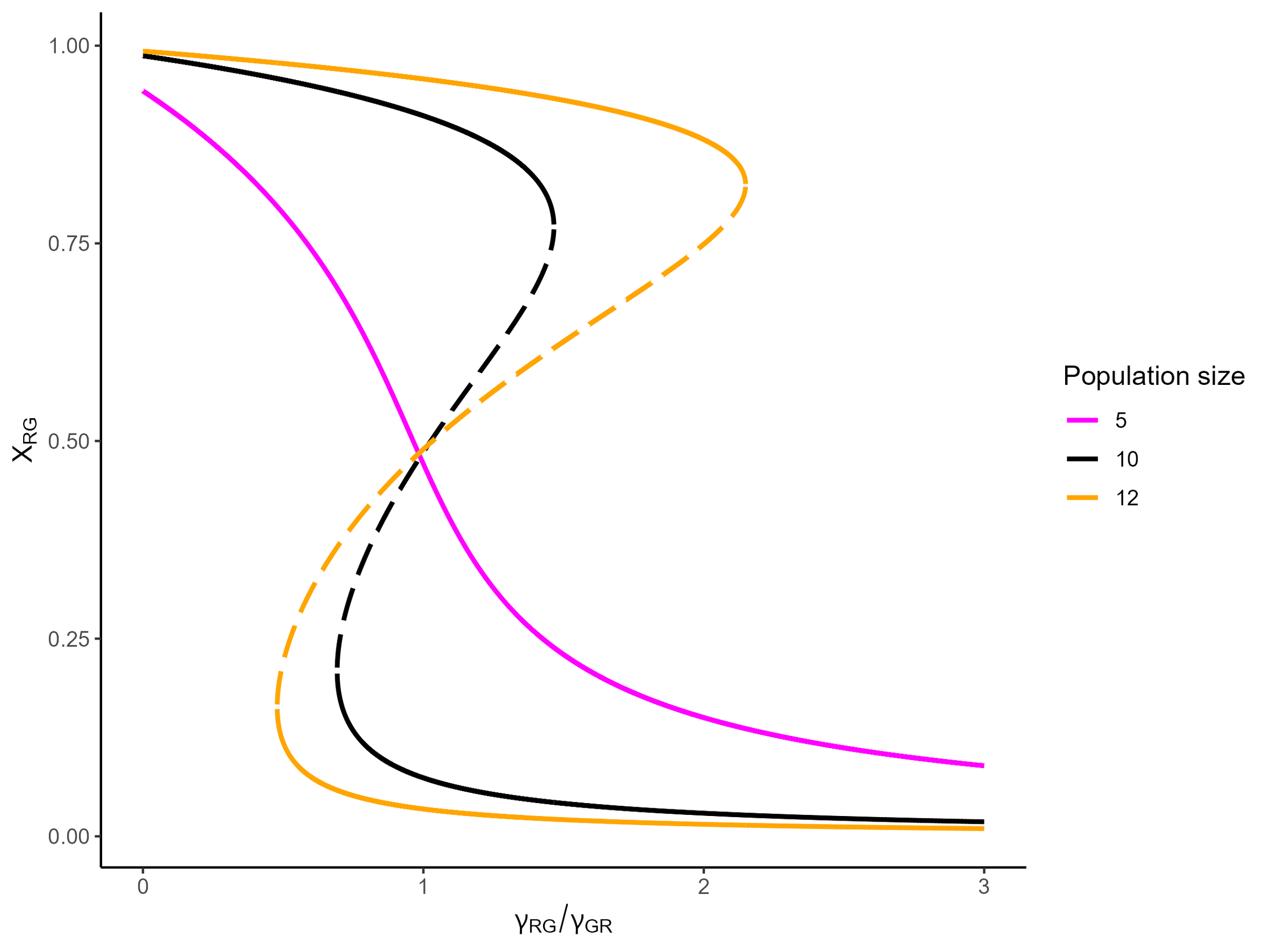

Supplement: S9 Fig — (TIF) [file pone.0287845.s009.tif]

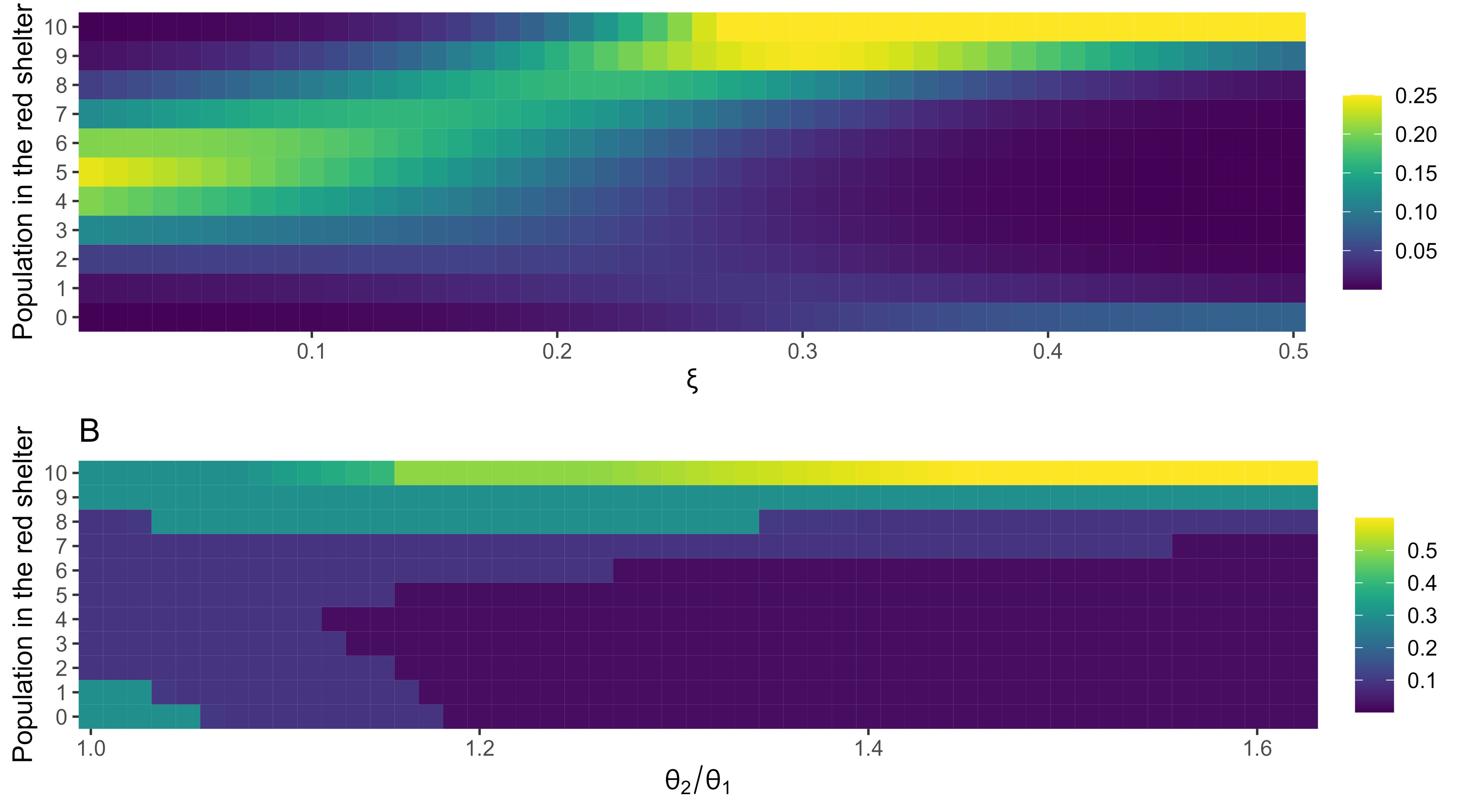

Supplement: S10 Fig — Integration from eq. S6a-S6c in S1 File. (TIF) [file pone.0287845.s010.tif]
