# Supplementary material for: Emergence and retention of a collective memory in cockroaches
Source: PLoS One. 2023 Jul 6;18(7):e0287845. doi: 10.1371/journal.pone.0287845 (PMC10325095; doi:10.1371/journal.pone.0287845)
Supplement: S1 File — (DOCX) [file pone.0287845.s011.docx]

# Supporting material

## Materials and methods

### Experimental set-up


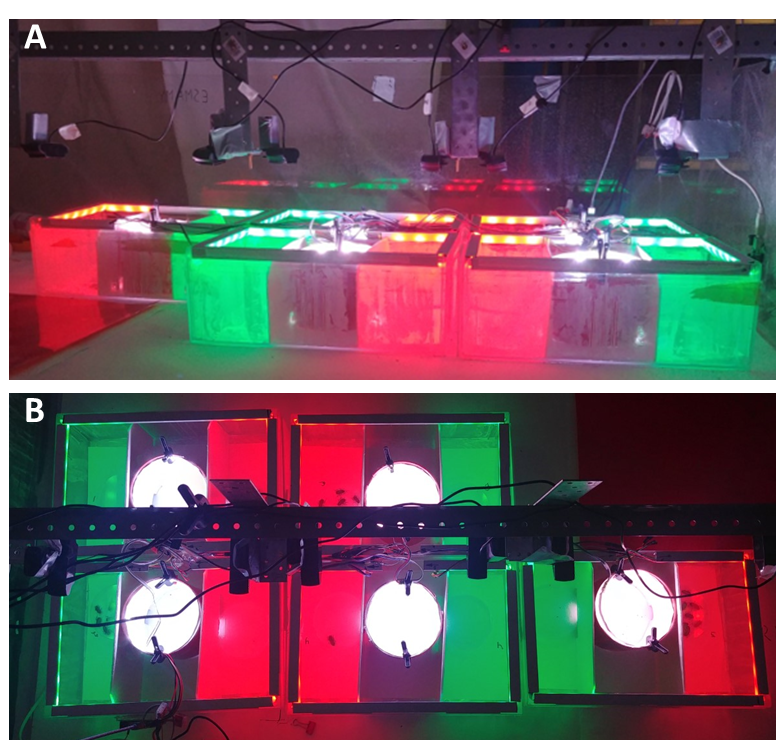


**Fig. S1: Experimental set-up.** A) Lateral view. B) Upper view*.*

Cameras are positioned between two experimental devices as shown on Fig. S1-2. This allows to record simultaneously 5 trials (set-up) with 6 cameras.


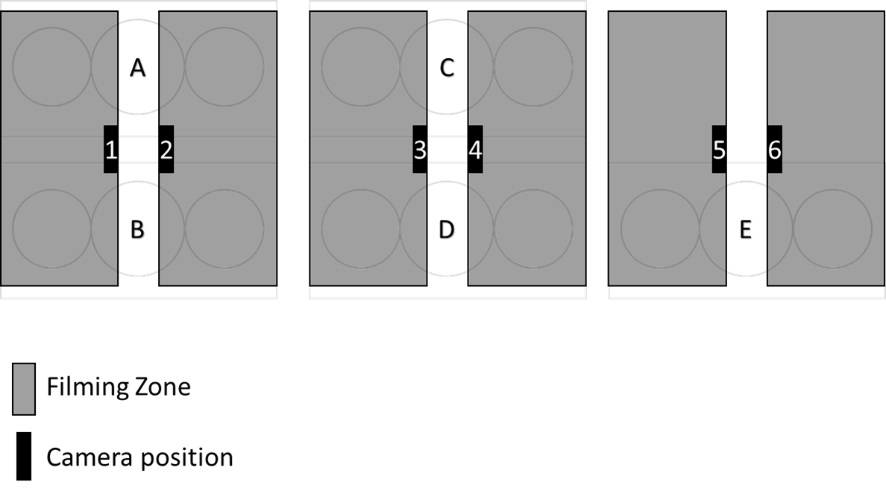


**Fig. S2: Camera position and filming zone**

### Data and statistical analysis

**Permutation and resampling tests**

Eq. S1 shows the number of possible combinations C(n,s) used for the complete combination method, where n is the size of the set of trials and s is size of the sample of trials. With s ≤ n

$$C\left( n,s \right)= \frac{n!}{s!\left( n-s \right)!} (S1)$$

Eq. S2 shows the number of possible permutation *P(n,s)* used for the complete permutation method where *n* is the number of shelters to permute (in our case 2) and *s* is the size of the sample of trials

$$P\left( n,s \right)=n^{s} (S2)$$

## Results

### Global sheltering process

The global sheltering process for each condition shows that for the control condition at 30 minutes the mean ± SEM proportion of total sheltered individuals is 0.6 ± 0.24 for the isolated individuals and is 0.78 ± 0.1 for the groups. Linear regressions of the total sheltered proportion of individuals over-time (between times 30 and 1320 minutes, fig S3) are not significant neither for the isolated individuals (N=5; R² = 5x10^-5^, F = 0.01, P = 0.91 – Intercept = 0.7, t = 11.16, P < 2x10^-16^ – Slope = -9.45x10^-6^, t = 0.11, P = 0.9) nor for the groups (N=5; R² = 0.007, F = 2.67, P =0.1 – Intercept = 0.86, t = 53.53, P < 2x10^-16^ – Slope= 3.4x10^-5^, t =1.6, P = 0. 1).

For the inversion condition at 30 minutes the mean ± SEM proportion of total sheltered individuals is 0.76 ± 0.1 for the isolated individuals and 0.94 ± 0.01 for the groups. The time (between 30 minutes and 1320 minutes, Fig S3) does not influence significantly the total sheltered proportion for the isolated individuals (N=21; R² = 0.0018, F = 2.79, P = 0.09 – Intercept = 0.68, t = 23.3, P < 2x10^-16^ – Slope = 6.4x10^-5^, t = 1.67, P = 0.09). For the groups and for the same time period, a linear regression of the total sheltered proportion of the population over-time shows a significant positive influence of the time (N=22; R² = 0.007, F = 8.7, P = 0.003– Intercept = 0.88, t = 122.2, P < 2x10^-16^ – Slope= 2.79x10^-5^, t =2.9, P < 0.003).


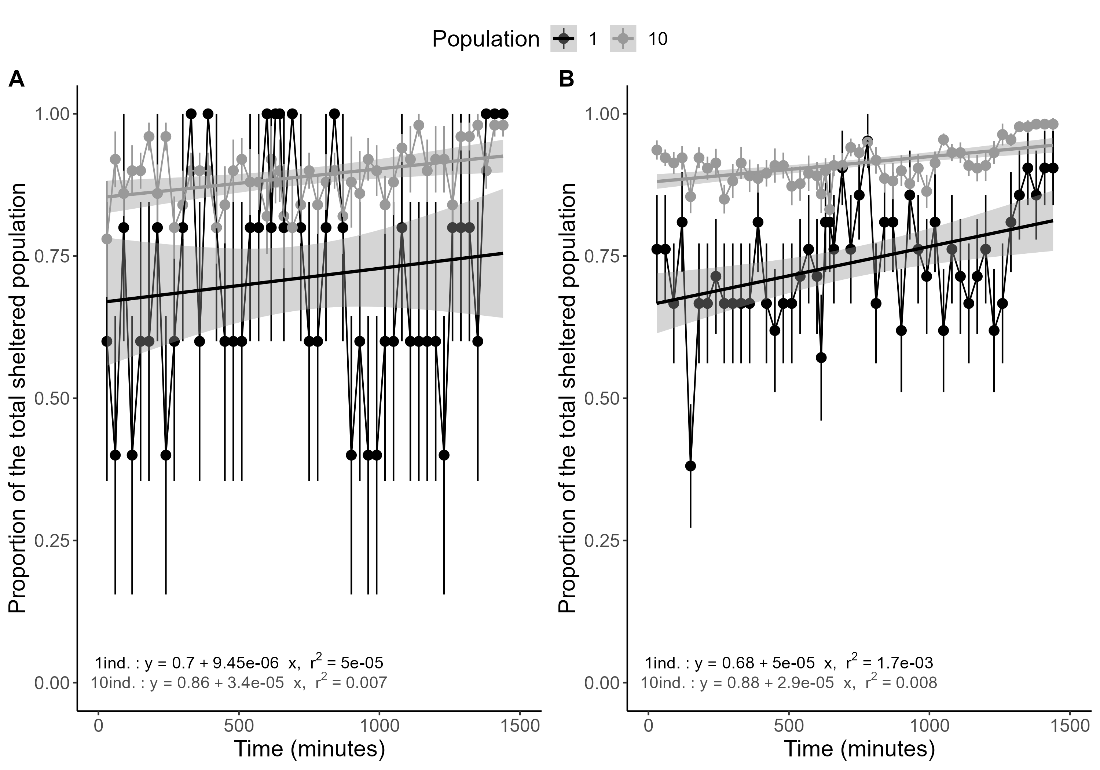


**Fig. S3: Sheltering process of the total population over time.** Mean ± SEM of isolated individuals (black) and of groups (grey). A) Control condition: Isolated individuals N = 5; groups N = 5. B) Inversion condition: isolated individuals N = 21; groups N = 22.

### Shelter colour influences

For the first 600 minutes the mean ± SEM cumulative presence (over 21 time-steps) of isolated individuals inside the red shelter for the control condition (10.2 ± 2.15) and the inversion condition (7.8 ± 1.04) were not significantly different (see section Statistical analysis and table 1). At every time-steps similar results are observed as well, except for four time-steps (table 1). For the groups the same tests show no significant difference for the mean ± SEM cumulative presence (over 21 time-steps) of individuals in the red shelter (table 1) between the control condition (134.4 ± 4.91) and the inversion condition (134.7 ± 6.22) and for every time-step, except for one time-step (table 1 in the main text). These results, allow us to pool the control and the inversion conditions for each population size, for the firsts 600 minutes.

Between times 660 and 1320 minutes the cumulative presence of isolated individuals in the red shelter (mean ± SEM) is not significantly different between the control and inversion conditions (table 1). Similarly, their presences for the control and for the inversion conditions, at every time-step, are not significantly different, except at three time-steps (table 1). Therefore, we pooled as well, the control and the inversion condition for the isolated individuals. The cumulative presence (mean ± SEM) inside the red shelter (12.19 ± 0.76) is significantly higher (table 1) than the one inside the green shelter (4.11 ± 0.34). However, there are significant differences between the control and the inversion conditions for the groups (table 1). Therefore the comparison between the shelter colours is made using only the inversion condition (see main text). Nonetheless, for the control condition the cumulative occupation (mean ± SEM) in the red shelter is greater than in the green shelter (Fig S4).

### Population influences

Concerning the sheltering behaviour differences between isolated individuals and groups, the results show that before the first 600 minutes for the pooled data (N_isolated individuals_ = 26; N_groups_ = 27) the cumulative proportion of the total population in the red shelter is significantly greater for the groups (table 1 in the main text). This result is less marked when considering at every time-step: only 7 of 21 time-steps are significantly different (table 1). Between times 660 and 1320 minutes for the inversion condition no significant difference is observed (table 1) for the cumulative proportion of the total population in the red shelter and at every time-step only 4 of 23 are significantly different (table 1).

**Table S1. Individuals distribution**

| Trial | RG shelter | GR shelter | Binomial test |
| --- | --- | --- | --- |
| 1 | 10 | 0 | P= 0.0019 |
| 2 | 10 | 0 | P= 0.0019 |
| 3 | 0 | 10 | P= 0.0019 |
| 4 | 0 | 10 | P= 0.0019 |
| 5 | 9 | 1 | P= 0.021 |
| 6 | 8 | 1 | P = 0.039 |
| 7 | 10 | 0 | P= 0.0019 |
| 8 | 1 | 8 | P = 0.039 |
| 9 | 0 | 10 | P= 0.0019 |
| 10 | 0 | 10 | P= 0.0019 |
| 11 | 0 | 10 | P= 0.0019 |
| 12 | 0 | 10 | P= 0.0019 |
| 13 | 0 | 9 | P = 0.0039 |
| 14 | 0 | 9 | P = 0.0039 |
| 15 | 0 | 10 | P= 0.0019 |
| 16 | 0 | 10 | P= 0.0019 |
| 17 | 1 | 9 | P= 0.021 |
| 18 | 0 | 10 | P= 0.0019 |
| 19 | 10 | 0 | P= 0.0019 |
| 20 | 0 | 10 | P= 0.0019 |
| 21 | 0 | 10 | P= 0.0019 |
| 22 | 10 | 0 | P= 0.0019 |

Consensus selection. Sheltered population at 1320 minutes for the group inversion condition and their respective binomial test between the wining shelter and the total sheltered population.


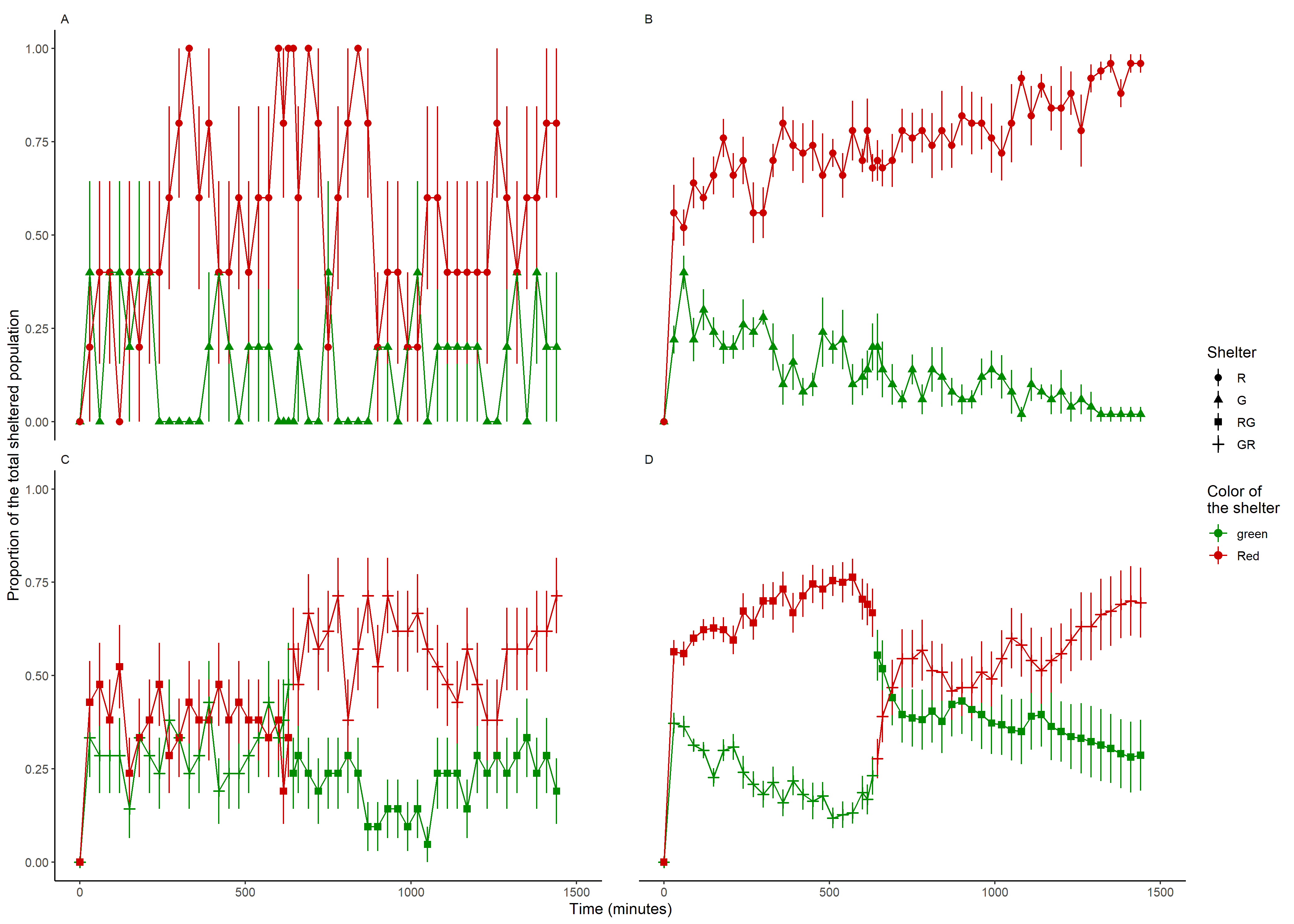


**Fig. S4: Sheltered population over time.** Mean ± SEM in the R shelter (dot), the G shelter (triangle), the RG shelter (square) and the GR shelter (cross). A) Control condition: Isolated individuals N = 5. B) Control condition: groups N = 5. C) Inversion condition: isolated individuals N = 21. D) Inversion condition: groups N = 22.


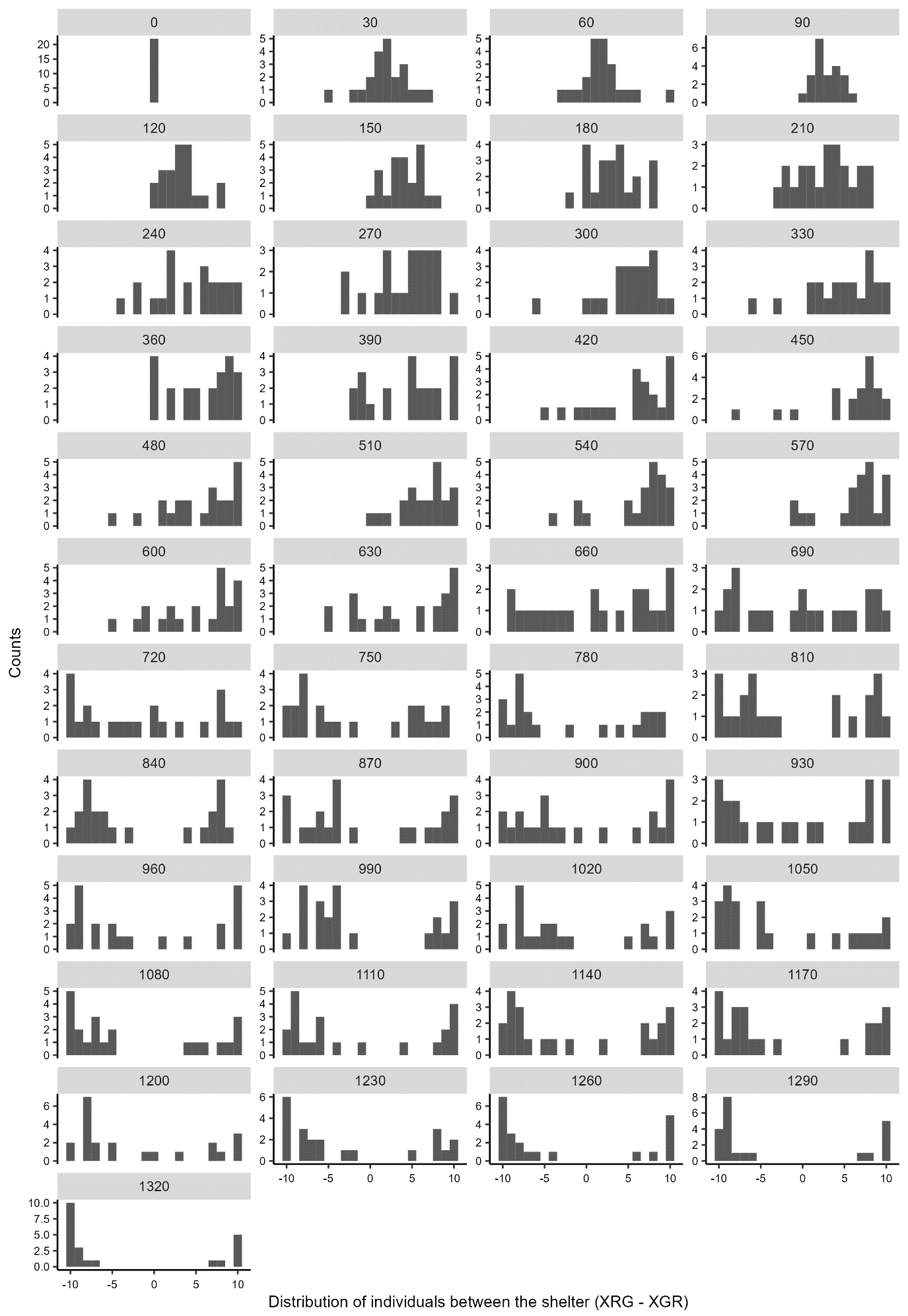


**Fig. S5: Group inversion condition: distribution of individuals between the shelter (XRG - XGR) for every time-step.** XRG : number of individuals in the RG shelter ; XGR : number of individuals in the GR shelter.


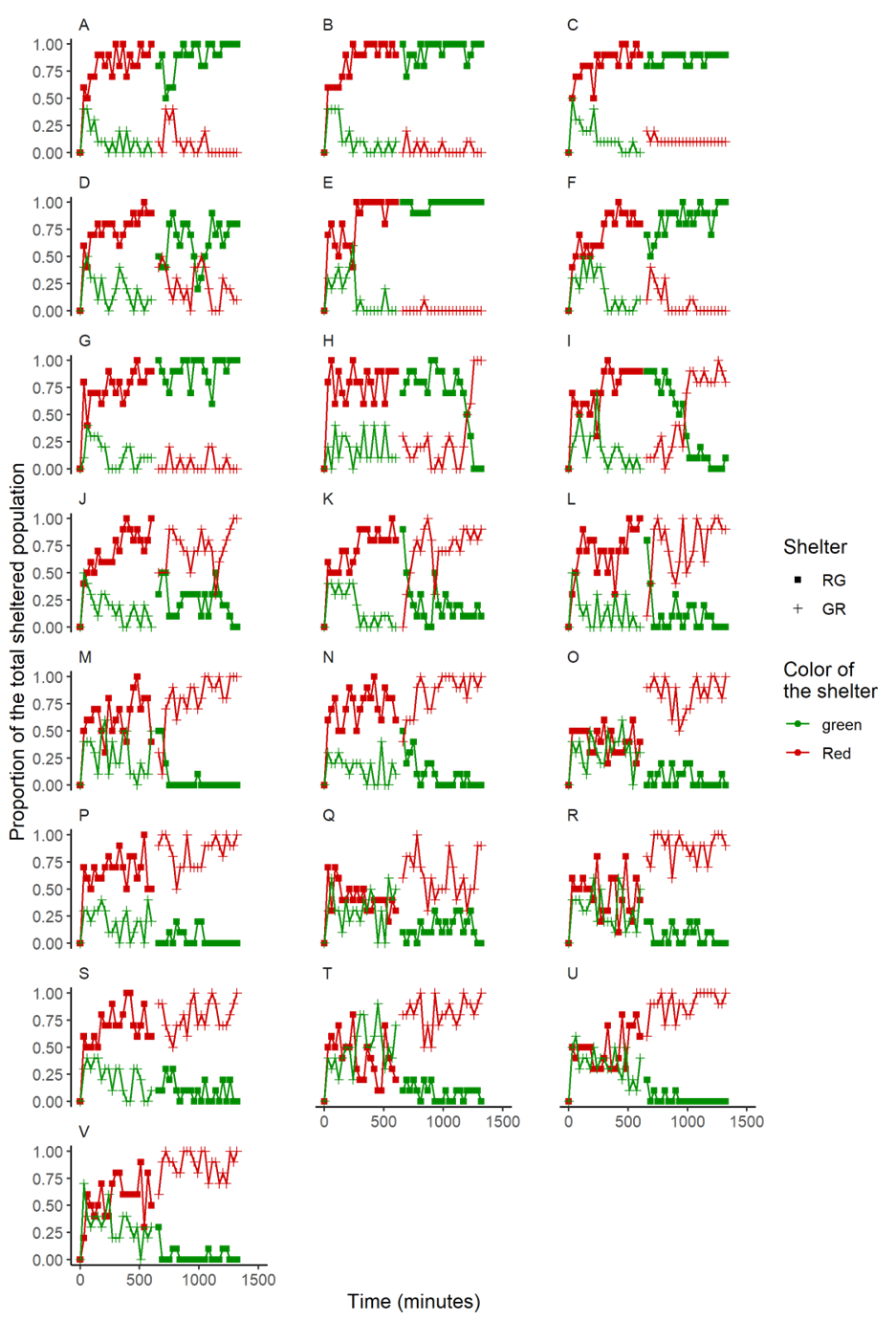


**Fig. S6: Proportion of the total population sheltered in the RG shelter (square) and in the GR shelter (cross) over-time.** A-G) Consensual groups in the RG at 600 and at 1320 minutes. H-L). Consensual groups in the RG at 600 minutes and in the GR at 1320 minutes. M-V) Non-consensual groups in neither shelter at 600 but consensual groups in the GR shelter at 1320 minutes. Colours indicate the luminosities of the shelters (red = dark shelter and green = light shelter) at each time-step. Each subplot is a trial.

### Mathematical model

**Deterministic view**

Several models have been developed and validated to establish the link between individual decisions and collective choices [1–5]. The dynamics of these collective choices lead typically to multiple steady states, among which stable states that are separated by unstable ones acting as thresholds [6,7]. In the case of sheltering dynamics, these models follow the same structure: the probabilities of joining and leaving each shelter may depend on the shelter characteristics and on the number of individuals already sheltered [5,8,9] To explain the emergence of collective choices, positive loops must be present, either by recruitment or attraction, the probability of joining increasing with the sheltered population and/or the probability of leaving decreasing with the occupancy rate. This leads to the following differential equations for the evolution of the number of individuals on shelter $i$ (in our case, $i=1,2$) through time:

$$\frac{dX_{i}}{dt}=N_{e}\mu_{i}f\left( X_{i} \right)-\theta_{i}X_{i}g\left( X_{i} \right) i=1,2 \left( S3a \right)$$

where $N_{e}$ is the number of unsheltered individuals, equal to $N-X_{1}-X_{2}$ and $\mu_{i}$ is the individual rate or probability of joining the shelter $i$, modulated in some cases by a function $f\left( X_{i} \right)$ depending on the number of individuals already settled in shelter $i$. In the present study, there are no evidence of any attraction of the sheltered individuals towards unsheltered individuals and therefore $f\left( X_{i} \right)=1$ for both shelters. Note also that interactions between cockroaches occur primarily over short distances. Indeed, the two main types of interactions are mediated by physical and chemical (cuticular hydrocarbons) contacts (10-13). The latter, present on the animal or deposited on the substrate, are mostly long chains with low volatility [10]. As for $\theta_{i}$, it is the maximal rate or probability of leaving the shelter $i$, modulated by a decreasing function $g\left( X_{i} \right)$with the number of individuals already settled in shelter $i$. Several functions $g\left( X_{i} \right)$ are described in the literature but leading to similar dynamics. Keeping in mind the Occam’s razor and based on previous works, we used an exponential function, a one-parameter function [4,5]:

$$g\left( X_{i} \right)=e^{-\xi X_{i}} \left( S3b \right)$$

where $\xi$ is the degree of cooperativity/interattraction between individuals inside the shelters, which is taken independent of the shelter quality. Labelling respectively the proportion of individuals in the red and in the green shelter as $x_{1}(=\frac{X_{1}}{N})$ and $x_{2} (=\frac{X_{2}}{N})$, and putting eqs. (S3a-b) at the stationary state ($\frac{dX_{i}}{dt}=0$) yields:

$$\begin{matrix} 0 & =\gamma_{1}\left( 1-x_{1}-x_{2} \right)-x_{1} e^{-\zeta x_{1}} \\ 0 & =\gamma_{2}\left( 1-x_{1}-x_{2} \right)-x_{2} e^{-\zeta x_{2}} \left( S4 \right) \end{matrix}$$

where $\gamma_{1}=\frac{\mu_{1}}{\theta_{1}}$, $\gamma_{2}=\frac{\mu_{2}}{\theta_{2}}$ and $\zeta=\xi N$. We end up therefore with the following expressions for $x_{1}$ and $x_{2}$ which depend on only three parameters

$$\begin{matrix} 0 & =\frac{\gamma_{1}}{\gamma_{2}}-\frac{x_{1}e^{\zeta\left( 1-2 x_{1}- \frac{x_{1}e^{\left( -\zeta x_{1} \right)}}{\gamma_{1}} \right)}}{1-x_{1}-\frac{x_{1}e^{\left( -\zeta x_{1} \right)}}{\gamma_{1}}} \\ x_{2} & =1-x_{1}-\frac{x_{1} e^{-\zeta x_{1}}}{\gamma_{1}} \left( S5 \right) \end{matrix}$$

Fig S7. displays the stable and unstable steady states of eq. S5 as a function of the parameter $\zeta$ for different values of $\gamma_{1}$ and different ratios $\frac{\gamma_{1}}{\gamma_{2}}$, They were obtained using numerical methods (Newton-Raphson). For a small value of $\gamma_{1}$ and identical shelters $\gamma_{1}=\gamma_{2}$ (Fig S7A), the maximum number of solutions is 7, among which three are stable. For small values of $\zeta$, corresponding to a weak interattraction or a small number of individuals in the setup, only one homogeneous solution exists which corresponds to a weak occupation of both shelters. As $\zeta$ increases, the homogeneous solution starts to coexist with two stable inhomogeneous solutions, corresponding to a unique aggregate in shelter 1 or 2. Increasing $\zeta$ further leads to a loss of stability of the homogeneous solution, leaving the system with the asymmetric solutions. When the quality of the shelters become different (Fig S7B-C), the situation remains qualitatively the same, except that the stable homogeneous solution is replaced by a weak inhomogeneous one. The situation changes slightly when increasing significantly $\gamma_{1}$ and fixing it to its experimental value (see adjustment section below). Indeed, when $\gamma_{2}=\gamma_{1}$ (Fig S7D), the homogeneous solutions corresponding to an equal occupation of both shelters with almost all individuals sheltered exists for small values of $\zeta$, and becomes unstable at a particular value of the parameter, where two inhomogeneous solutions take place. Again, the situation is almost identical for different $\gamma_{2}$ and $\gamma_{1}$(Fig S7E-F), where the homogeneous solution is replaced by a weak inhomogeneous one for small values of $\zeta$, the difference with the previous case of a small value of $\gamma_{1}$ being that almost all individuals are this time sheltered.


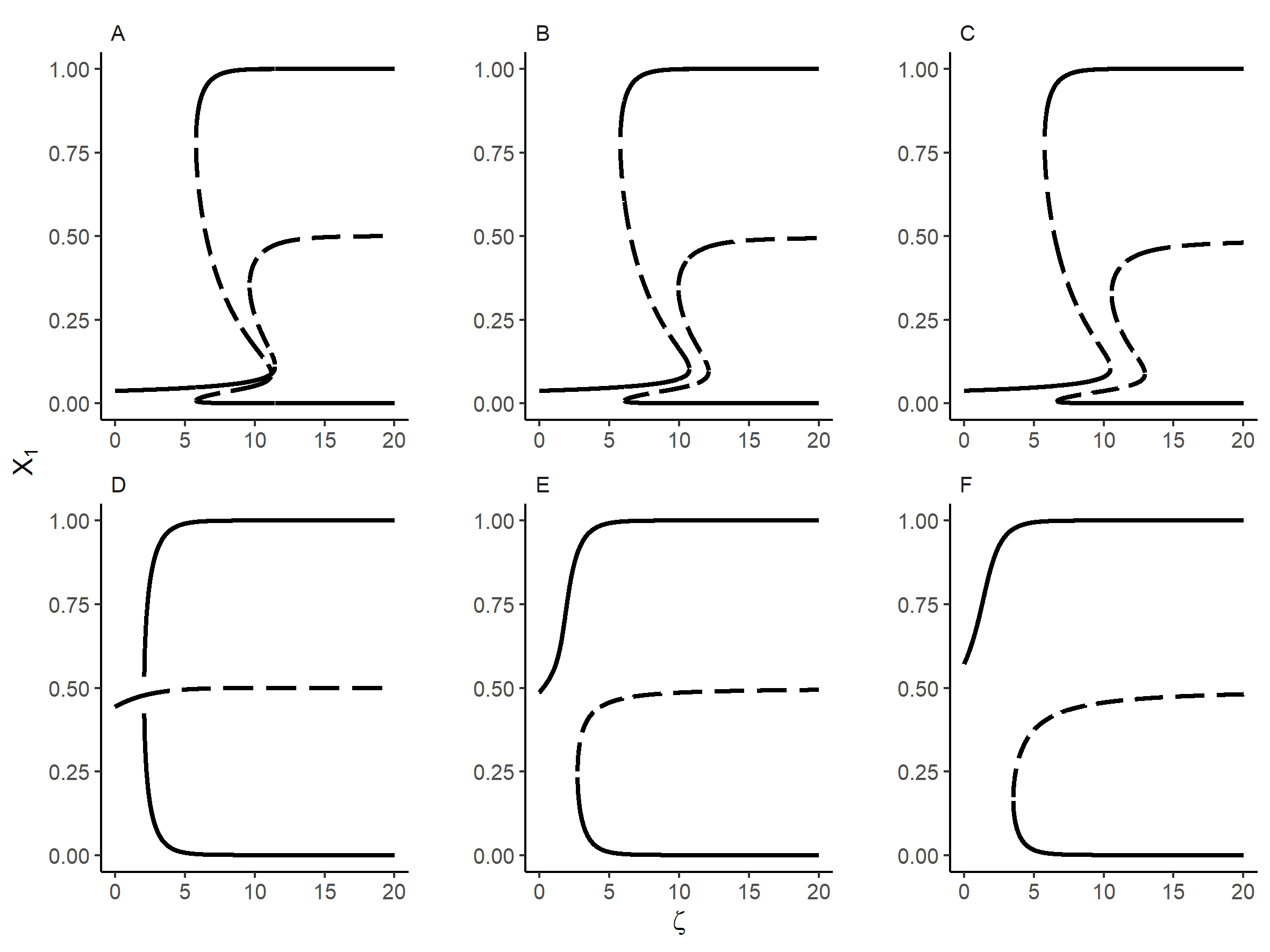


**Fig. S7: Bifurcation diagrams of the stationary solutions of the eq. S5 as a function of ζ.** For symmetrical (A; D) and asymmetrical (B - C; E – F) shelter qualities. A) γ_1_ = γ_2_ = 0.04. B) γ_1_ = 0.04; γ_2_ = 0.032. C) γ_1_ =0.04; γ_2_ = 0.02. D) γ_1_ = 4; γ_2_ = 4. E) γ_1_ = 4; γ_2_ = 3.2. F) γ_1_ = 4; γ_2_ = 2*.*

A more global view is displayed in Fig. S8 where all the parameters space is explored for different ratios $\frac{\gamma_{2}}{\gamma_{1}}$ (1, 0.5 and 0.8). They confirm that the different qualities of the shelter do not change qualitatively the patterns of decision-making and that the available solutions are controlled mainly by the number of individuals involved and the strength of the interattraction ($\zeta=\xi N$), and by the ratio of the probabilities to join and to leave a shelter ($\gamma_{i}=\frac{\mu_{i}}{\theta_{i}},i=1,2$).


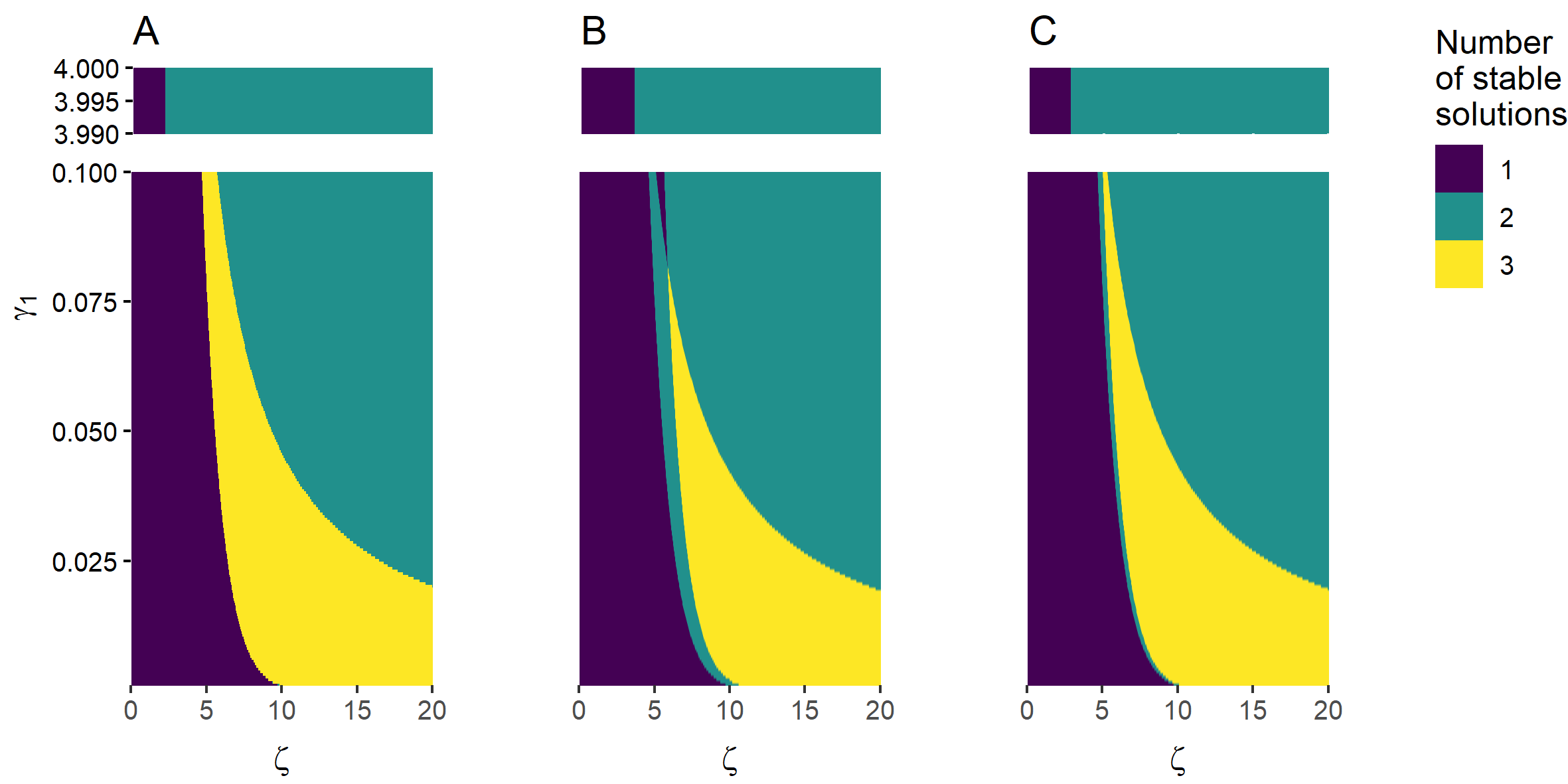


**Fig. S8 Number of stable solutions given by eq. S5 as a function of gamma and zeta.** A) Symmetrical shelter qualities γ_2_ = γ_1_. B) Asymmetrical shelter qualities γ_2_ = 0.5 γ_1_. C) Asymmetrical shelter qualities γ_2_ = 0.8 γ_1_

As far as our experiments are concerned, a clear view is provided by Fig S9, where the steady state solutions are displayed as a function of $\frac{\gamma_{2}}{\gamma_{1}}$ for different values of $\zeta$ ($\zeta= \xi N$). Keeping $\xi$ constant it shows that beyond a value of $N$, we go from a monotonic dependence of $\frac{\gamma_{2}}{\gamma_{1}}$, where the number of sheltered individuals on the red shelter decreases as $\frac{\gamma_{2}}{\gamma_{1}}$ increases; to a situation where hysteretic behaviours emerge for larger $N$. The onset of a collective memory is therefore only possible beyond a critical value of the group size (or a strong interattraction).


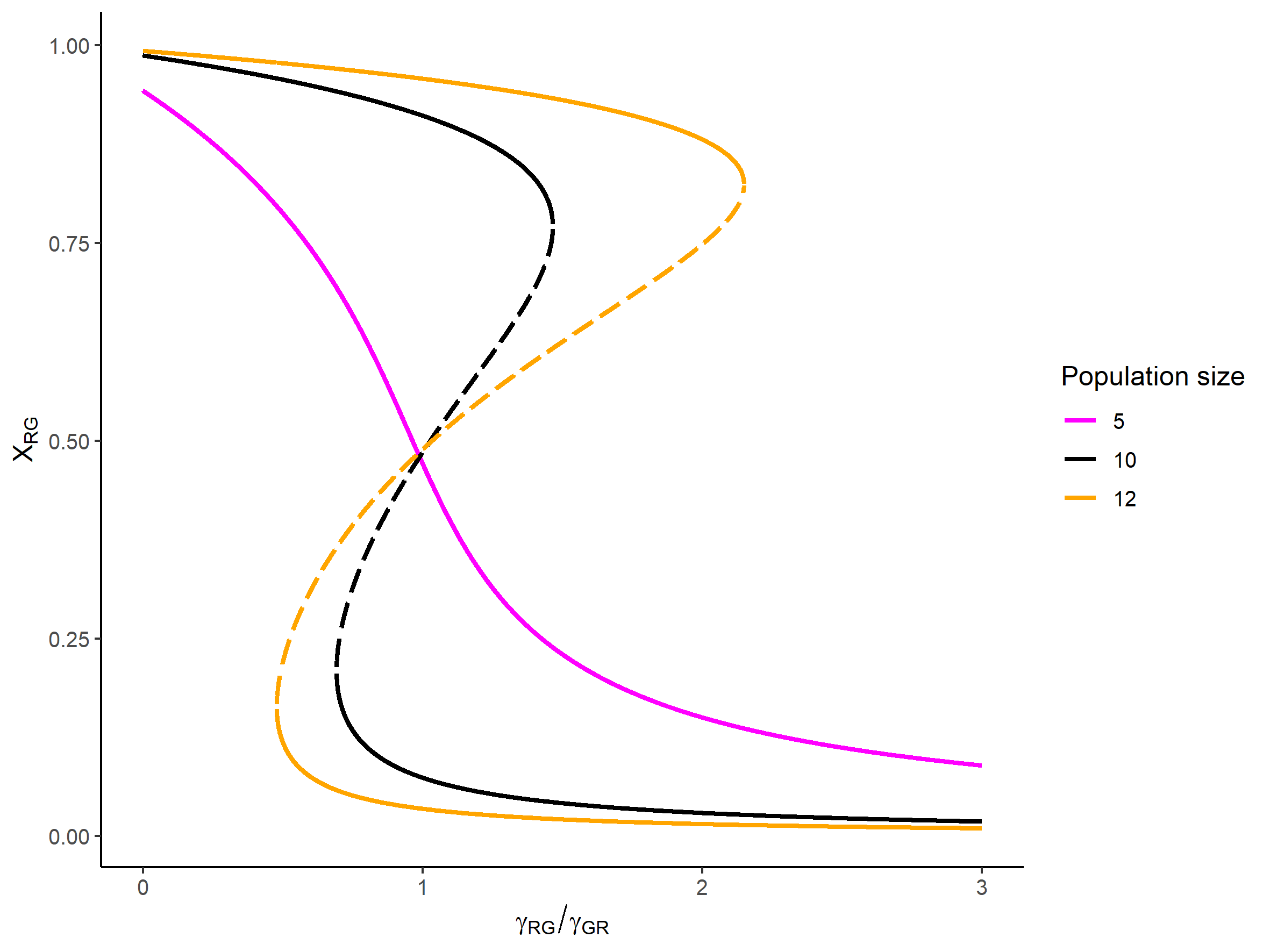


**Fig S9: Bifurcation diagram of eq. S5.** Lines corresponding to the stable (solid line) unstable (dashed line) steady states of the proportion of the population in the RG as a function of $\frac{\gamma_{2}}{\gamma_{1}}$ for groups of 5 (magenta), 10 (black) and 12 (orange) individuals. Parameters values: γ_1_ = 4; γ_2_ range from 0.001 to 12. Other parameter values $\zeta=0.3$

**Stochastic model: master equation**

In order to take into account, the stochasticity in the phenomenon of interest, which for small number of individuals play an important role concerning the transition between the different states, we will use the stochastic version of the model described in eq. S3: the master equation. This consists of a set of first-order differential equations which describes the time evolution of the probability $P\left( X_{1},X_{2} \right)$ of the system to occupy each of the discrete sets of states taken by the system. $X_{1}$ and $X_{2}$ can take any values between 0 and $N$ ($N$ being the total number of individuals) and the evolution of $P\left( X_{1},X_{2} \right)$ over time depends on the transitions $W$ leading to this state or removing from it. To each birth and death transition corresponds a transition probability, equals to the individual probability to perform a behaviour (joining, leaving) times the number of individuals able to perform the corresponding behaviour. Note finally that in the stochastic version of the model eq. S3a, the leaving probability given by eq. S3b is changed to take into account the fact that an individual sheltered is not counting itself in the process, i.e.:

$$g\left( X_{i} \right)=e^{-\xi\left( X_{i}-1 \right)} \left( S5 \right)$$

The master equation is the following set of differential equations:

$$\begin{matrix} \frac{dP\left( X_{1},X_{2} \right)}{dt} & =W\left( X_{1}+1,X_{2}\to X_{1},X_{2} \right)P\left( X_{1}+1,X_{2} \right) \\ & +W\left( X_{1},X_{2}+1\to X_{1},X_{2} \right)P\left( X_{1},X_{2}+1 \right) \\ & +W\left( X_{1}-1,X_{2}\to X_{1},X_{2} \right)P\left( X_{1}-1,X_{2} \right) \\ & +W\left( X_{1},X_{2}-1\to X_{1},X_{2} \right)P\left( X_{1},X_{2}-1 \right) \\ & -(W\left( X_{1},X_{2}\to X_{1}-1,X_{2} \right)+W\left( X_{1},X_{2}\to X_{1},X_{2}-1 \right)+ \\ & W\left( X_{1},X_{2}\to X_{1}+1,X_{2} \right)+W\left( X_{1},X_{2}\to X_{1},X_{2}+1 \right))P\left( X_{1},X_{2} \right) \\ & X_{1},X_{2}=0,\cdots, N,X_{1}+X_{2}\leq N \left( S6a \right) \end{matrix}$$

Where the four birth transitions leading to the state $\left( X_{1},X_{2} \right)$ are:

$$\begin{matrix} W\left( X_{1}+1,X_{2}\to X_{1},X_{2} \right) & =\theta_{1}\left( X_{1}+1 \right)g\left( X_{1}+1 \right) \\ W\left( X_{1},X_{2}+1\to X_{1},X_{2} \right) & =\theta_{2}\left( X_{2}+1 \right)g\left( X_{2}+1 \right) \\ W\left( X_{1}-1,X_{2}\to X_{1},X_{2} \right) & =\mu_{1}\left( N+1-X_{1}-X_{2} \right) \\ W\left( X_{1},X_{2}-1\to X_{1},X_{2} \right) & =\mu_{2}\left( N+1-X_{1}-X_{2} \right) \left( S6b \right) \end{matrix}$$

and the four death transitions from the state $\left( X_{1},X_{2} \right)$ are:

$$\begin{matrix} W\left( X_{1},X_{2}\to X_{1}-1,X_{2} \right) & =\theta_{1}X_{1}g\left( X_{1} \right) \\ W\left( X_{1},X_{2}\to X_{1},X_{2}-1 \right) & =\theta_{2}X_{2}g\left( X_{2} \right) \\ W\left( X_{1},X_{2}\to X_{1}+1,X_{2} \right) & =\mu_{1}\left( N-X_{1}-X_{2} \right) \\ W\left( X_{1},X_{2}\to X_{1},X_{2}+1 \right) & =\mu_{2}\left( N-X_{1}-X_{2} \right) \left( S6c \right) \end{matrix}$$

Moreover, $\Pi\left( X_{i} \right)$ is the probability to have $X_{i}$ individuals the shelter $i$, independently of the population sheltered in the second shelter or on the individuals outside the shelters.

$$\Pi\left( X_{i} \right)=\sum_{X_{j}=0}^{N-X_{i}} P\left( X_{i},X_{j} \right) {i=1,2 ; j=2,1 ; X}_{i}, X_{j}=0,\cdots N (S7)$$

The stationary probability distribution is obtained by integrating the equations (S5a-c) and is either mono or multimodal. It gives the probabilities of the system to reach all configurations $\left( X_{i},X_{j},i=0,\ldots,10,j=0,\cdots,10 \right)$. In our case, a bimodality at the stationary states corresponds to the existence of two distinct stable collective choices.

**Adjustment**

We numerically integrate, with a time-step of 1 second, the master eq. S6 (and calculate eq. S7) for 630 minutes and with an initial state simulating the beginning of the trials (X_1_ = 0 and X_2_ = 0 corresponding to all individuals outside the shelters), $\theta_{1}$ and $\theta_{2}$ respectively correspond to the red (RG) and the green shelter (GR). After 630 minutes, while keeping the state probabilities *P(X1, X2)* obtained from the first integration we invert the parameters value $\theta_{1}$ and $\theta_{2}$ and then integrate the same equation for 690 minutes. Using numerous combinations of parameter values we compare the theoretical results with the experimental ones to identify the parameter values that gives theoretical results compatible with the experimental ones.

In our settings, we assume for simplicity that the ratio $\mu_{1}/\mu_{2}$ is equal to the ratio $\theta_{2}/\theta_{1}$ and that all individuals behave in the same way, i.e., no idiosyncrasy is at work.

$$\mu_{1}=\frac{\mu\theta_{2}}{\theta_{1}+\theta_{2}};\mu_{2}=\frac{\mu\theta_{1}}{\theta_{1}+\theta_{2}} (S8)$$

The goodness-of-fit of the model is tested using the Hellinger distance (HD) between the theoretical distribution P(X_i_) and the experimental one. The test consists in 20000 random sampling of 22 observations of the theoretical distribution. We calculate for each sampling the HD statistic, then once this process is completed, we evaluate how many times the experimental statistic exceeds the theoretical one (at t = 600 minutes and at t = 1320 minutes). The p-value is thus deduced. Numerous combinations of parameters values are tested:

$$\mu=0.002,0.0022,\ldots,0.01 ;\xi= 0.1,0.11,\ldots,0.5$$

$$\theta_{\mathrm{red}}=0.0001, 0.0002,\ldots,0.005; \theta_{green}=\theta_{\mathrm{red}}+0.0001, +0.0002,\ldots+0.005$$

The combination of parameters that yield the bests P values form the goodness of fit retained are shown in S2 table and used to calculate the mean ± sd of each parameter shown in the main text.

**Table S2. Parameters adjustment**

| *μ s^-1^* | θ_red_ *s^-1^* | θ_green_ *s^-1^* | *ξ* |
| --- | --- | --- | --- |
| 6.7x10^-3^ | 8 x10^-4^ | 9 x10^-4^ | 0.302 |
| 6.9 x10^-3^ | 8 x10^-4^ | 9 x10^-4^ | 0.306 |
| 7 x10^-3^ | 8 x10^-4^ | 9 x10^-4^ | 0.306 |
| 7.1 x10^-3^ | 8 x10^-4^ | 9 x10^-4^ | 0.302 |
| 7.2 x10^-3^ | 8 x10^-4^ | 9 x10^-4^ | 0.304 |
| 7.3 x10^-3^ | 8 x10^-4^ | 9 x10^-4^ | 0.3 |
| 7.3 x10^-3^ | 8 x10^-4^ | 9 x10^-4^ | 0.304 |
| 7.3 x10^-3^ | 8 x10^-4^ | 9 x10^-4^ | 0.306 |
| 7.4 x10^-3^ | 8 x10^-4^ | 9 x10^-4^ | 0.304 |
| 7.5 x10^-3^ | 8 x10^-4^ | 9 x10^-4^ | 0.304 |
| 7.5 x10^-3^ | 8 x10^-4^ | 9 x10^-4^ | 0.306 |
| 7.6 x10^-3^ | 8 x10^-4^ | 9 x10^-4^ | 0.3 |
| 7.6 x10^-3^ | 8 x10^-4^ | 9 x10^-4^ | 0.304 |
| 7.6 x10^-3^ | 8 x10^-4^ | 9 x10^-4^ | 0.306 |
| 7.7 x10^-3^ | 8 x10^-4^ | 9 x10^-4^ | 0.302 |
| 7.7 x10^-3^ | 8 x10^-4^ | 9 x10^-4^ | 0.306 |
| 7.9 x10^-3^ | 8 x10^-4^ | 9 x10^-4^ | 0.3 |
| 8 x10^-3^ | 8 x10^-4^ | 9 x10^-4^ | 0.304 |

**Parameters values obtain from the best goodness of fit of the adjustment of eq. S6-S7 to the experimental data.**


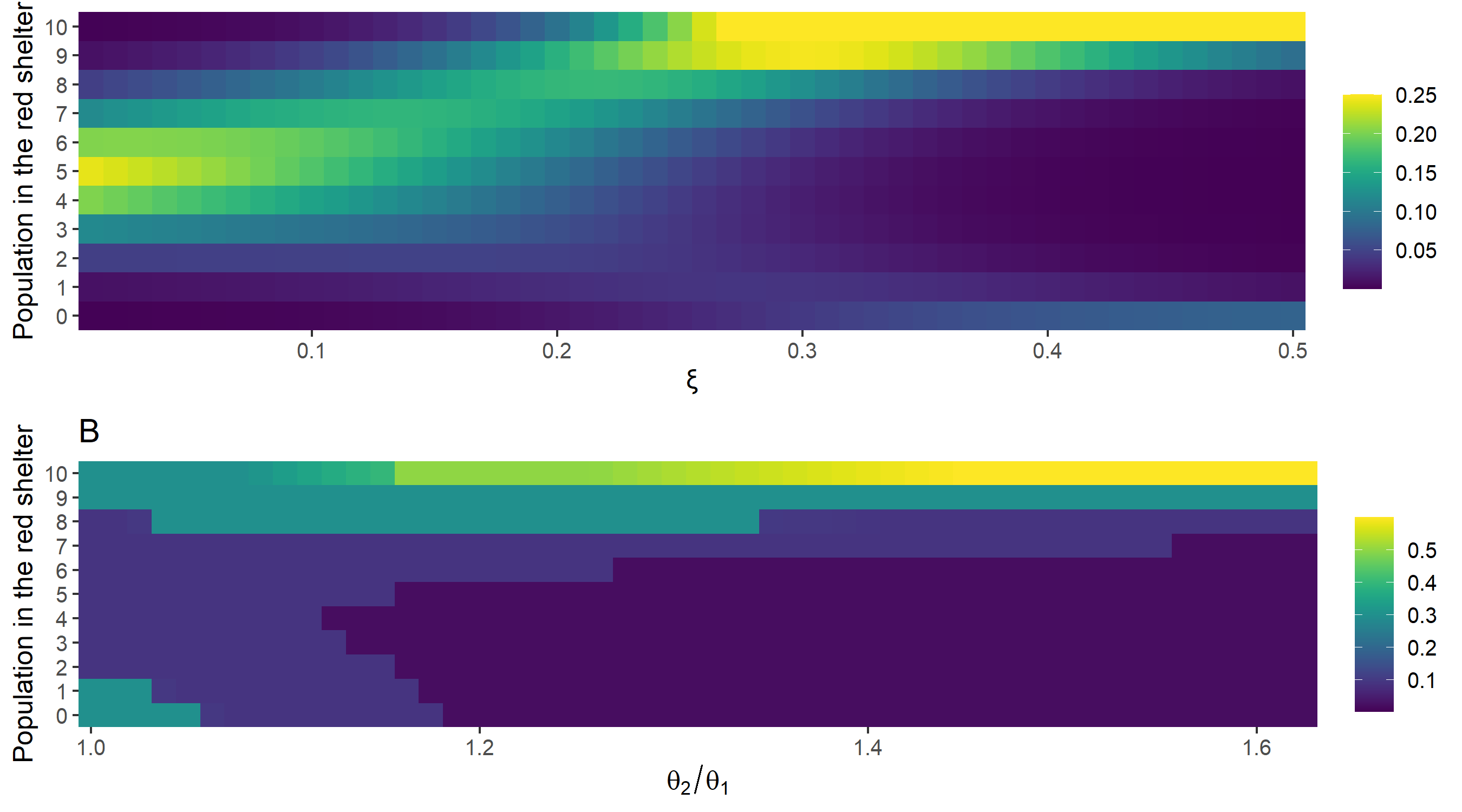


**Fig. S10 Sheltered population in the RG shelter at the steady state.** integration from eq. 6a-c. Total population size N = 10. A) Influence of the interattraction (ξ) (parameters values: θ_r_ = 8x10^-4^ s^-1^ and θ_g_ = 9x10^-4^ s^-1^) B) Influence of the shelter qualities (parameter value: ξ = 0.302). Other parameters values: µ_r_ = 3.55x10^-3^ s^-1^, µ_g_ = 3.15x10^-3^ s^-1^.

### References

1. Jeanson R, Dussutour A, Fourcassié V. Key Factors for the Emergence of Collective Decision in Invertebrates. Front Neurosci. 2012;6: 121. doi:10.3389/fnins.2012.00121

2. Sumpter DJT. Collective Animal Behavior. Princeton NJ US, editor. Collective Animal Behavior. Princeton: Princeton University Press; 2010. doi:10.1515/9781400837106

3. Amé J, Halloy J, Rivault C, Detrain C, Deneubourg JL. Collegial decision making based on social amplification leads to optimal group formation. Proc Natl Acad Sci. 2006;103: 5835–5840. doi:10.1073/pnas.0507877103

4. Calvo Martín M, Nicolis SC, Planas-Sitjà I, Deneubourg J-L. Conflictual influence of humidity during shelter selection of the American cockroach (Periplaneta americana). Sci Rep. 2019;9: 20331. doi:10.1038/s41598-019-56504-w

5. Calvo Martín M, Eeckhout M, Deneubourg J-L, Nicolis SC. Consensus driven by a minority in heterogenous groups of the cockroach Periplaneta americana. iScience. 2021;24: 102723. doi:10.1016/j.isci.2021.102723

6. Nicolis G, Prigogine I. Self-organization in nonequilibrium systems : from dissipative structures to order through fluctuations. New York: Wiley; 1977.

7. Thomas R, D’Ari R. Biological Feedback. HAL CCSD. Hal-00087681, editor. CRC Press, Inc.; 1990. Available: https://hal.archives-ouvertes.fr/hal-00087681

8. Jeanson R, Deneubourg JL. Conspecific attraction and shelter selection in gregarious insects. Am Nat. 2007;170: 47–58. doi:10.1086/518570

9. Pogson M. Simulation of Invertebrate Aggregation Shows the Importance of Stable Personality over Diversity in Consensus Decision-Making. Ito E, editor. PLoS One. 2016;11: e0165082. doi:10.1371/journal.pone.0165082

10. Saïd I, Christian M, Virginie D, Colette R. Intraspecific Signals Inducing Aggregation in Periplaneta americana (Insecta: Dictyoptera). Environ Entomol. 2015;44: 713–23. doi:10.1093/ee/nvv035
