# Supplementary material for: Emergence and retention of a collective memory in cockroaches
Source: PLoS One. 2023 Jul 6;18(7):e0287845. doi: 10.1371/journal.pone.0287845 (PMC10325095; doi:10.1371/journal.pone.0287845)
Supplement: Attachment — Submitted filename: point by point.docx [file pone.0287845.s012.docx]

Dear Editor,

We thank you and the reviewer for the reading and the constructive comments on our paper.

Hoping that our manuscript “Emergence and retention of a collective memory in cockroaches” is now suitable for publication in PLOS ONE, please find below a point by point response to the reviewer comments on our manuscript.

*Major comments.*

- *The choice of the exponentially decaying function with number of individuals in a zone that modulates the leaving rate is not justified enough. For a reader to understand the origin of this choice is crucial not only to appreciate the modelling part but to link it with the actual behaviours. Hence, I expect more justification by adding a paragraph on it. It took me some time and also I had to cross check the reference cited (Calvo Martín et al. 2019) to understand the motivation behind the choice.*

There is no doubt that the dynamics observed in this work involve positive feedbacks between individuals, which correspond to an attraction, a retention or both (see below) and are due to social inter-attractions between the individuals. They affect either the probabilities or rates of joining a site that increase with the population occupying this site or exit probabilities/rates that decrease with the population occupying that site. Several functions meet these conditions (e.g., Hill type function/threshold or exponential functions) [1–5]. There is also no doubt that these functions are approximations and it is not always easy to discriminate them experimentally. Therefore, having Occam's razor in mind and previous adjustments [6], the exponential function seems to be the best choice. Two versions of the model one with the threshold function and one with an exponential function give qualitatively the same collective behaviour (not shown here, it is not the objective of the present paper).

- *While, I mechanistically understand, how this choice can lead to the observed experimental results, it is yet unclear to me why this choice was made. For instance, a modulation of the joining rate with a similar function that increases exponentially with the number of individuals in that zone may also address the observation. (The authors claim that they find no evidence for attraction in their experiments, but it was unclear to me what is the basis of this claim.) Or, for that matter, both the modulation functions can depend on the number of individuals in a zone, however their relative strengths can be different. Hence, I think that this point could be addressed by making the model more general to modulate both the joining as well as leaving rate. More broadly, alternative models (at least one) and why they may as well or may not work should be checked and included in the text if possible. If the modelling is not possible, a small survey of literature of similar models should be incorporated.*

Regarding a probability/rate of joining modulated by the presence of conspecifics in the shelter, we totally agree. In fact, the general model including feedbacks in the joining and leaving rate was/is in the supplement. Two results (out of the scope of this paper) can be mentioned: whether there is a feedback on the input and a constant output rate or the opposite (as in this paper): a positive feedback on the leaving and a constant rate of joining, the two models exhibit similar behaviours. For example, equations 1 and 2 with $F\left( X_{i} \right)=\frac{1}{G(X_{i})}$ have the same stationary states and the same stability. The only difference is in the rate of evolution.

$$\begin{matrix} \frac{dX_{i}}{dt}= \mu\left( N-\sum_{i=1}^{2} X_{i} \right)-\theta X_{i} G(X_{i}) & & eq. 1 \\ Or & & \\ \frac{dX_{i}}{dt}= \mu F(X_{i})\left( N-\sum_{i=1}^{2} X_{i} \right)-\theta X_{i} & & eq.2 \\ with & & \\ i=1, 2 & & \end{matrix}$$

Furthermore, when both feedbacks (joining and leaving) are involved, there is a synergy between the two. Nonetheless, the predictions of the model remain qualitatively similar to those of the model with only one feedback. For example, in the case where *F(X_i_)* and *G(X_i_)* are exponential functions:

$$\begin{matrix} \frac{dX_{i}}{dt}={\mu e}^{\xi^{'}X_{i}}\left( N-\sum_{i=1}^{2} X_{i} \right)- X_{i}\theta e^{-\xi X_{i}} & & eq. 3 \\ & & \\ 0=\mu\left( N-\sum_{i=1}^{2} X_{i} \right)- X_{i}\theta e^{-(\xi+\xi^{'})X_{i}} & & eq.4 \end{matrix}$$

With $\xi^{'}$ and $\xi$ being the respectively the social strength of the joining and leaving feedbacks. he properties of eq. 4 (at the stationary state) are similar to those of eqs. 1 or 2.

In addition to these theoretical considerations, there are biological aspects to consider on the choice of the main mechanisms behind the observed dynamics. Indeed, in this article, based on previous experimental results, we chose to neglect the feedback on the joining process and to focus on the feedbacks related to the leaving probability/rate. Indeed, many studies have shown that the individual leaving rate decreases with the population inside the shelter [2,3,6], this mechanism of retention is facilitated on one hand through the thigmotactism and on the other hand through the cuticular hydrocarbons (CHCs) of the insects, being also deposited on the substrate [7–10]. The majority of these CHCs are long chains of hydrocarbons [11] and therefore have a low volatility and a low attractive effect (at short range) [9].

Although we do not exclude that a positive social feedback (substrate being marked by these CHCs) might increase the individual joining rate, this type of feedback is “weak” as compared to the feedback governing the leaving rate.

Please see the new version of the Mathematical model section of the supplementary.

*• The model does show hysteresis as a function of the individual choice for large group size. However, since this particular result hasn’t been shown in the experiments, the claim particularly about hysteresis should be toned down. A possible experimentation to show this could be inverting the light conditions in a more gradual or continuous manner. Two initial conditions for different group sizes might be used. One: keeping all individuals in the preferred zone at the start, and two: keeping all of them in the non preferred zone. A plot of the proportion of individuals in either of the zone as a function of changing lighting conditions might actually be able to show hysteresis properly in experiments. However, this, I understand, is definitely out of the current scope of the work. Therefore, I suggest to avoid using hysteresis. The authors have right now shown the existence of collective memory or inertia but just for an abrupt switch of the conditions which I think is insufficient to prove hysteresis.*

We fully agree with your description of hysteresis and the methodology to highlight it: two different initial conditions and gradual changes of parameters (assuming that for each new parameter value, the system reaches a stationary state). We agree that we do not show the classical hysteretic cycle or as you suggest to test with two initial conditions. Some of our statements can be disturbing and therefore, we followed your recommendation to use more cautiously the term hysteresis and to use mainly “bistability” and bimodality (already used in our first version). Just a brief remark: In a bifurcation diagram (states/values as a function of a bifurcation parameter), hysteresis is a bistability window (see below) between two zones of parameter values with only one stable state. But such bifurcation diagram can be misleading: the main characteristic of hysteresis can be maintained even with discontinuous and/or abrupt changes (irreversibility and the choice of a state according to the direction of the change of the bifurcation parameter).

*• The use of the stochastic version of the model was not clear to me. Basically, if the deterministic version is capable of capturing the observed trends, then the reasons to make it stochastic are not clear.*

We agree that the deterministic version captures most of the observed phenomena (e.g. stability of states, multiple states, …). But a stochastic version (stochastic equation) is able to give the theoretical frequencies of the different states of the system, necessary to make a valid theory-experiment comparison. In the cases of multistable systems if the deterministic analysis “assumes” that the system remains in the neighbourhood of a stable state, stochasticity can lead a system to leave the basin of attraction of a stable state and to join another basin. In other words, for the same initial conditions two replica can lead after a time to two different solutions and to a bimodal distribution. In the same vein, the stochastic version is able to highlight the presence of a transitory bimodality which is more cumbersome with the deterministic formulation.

*Minor comments.*

*I found several grammatical mistakes and also typos (especially in equations) that must be fixed. Some of the following points are also not really minor as they deal with the readability of the paper and are, therefore, very important for building an understanding while reading the text; While I think the experimentation and the ideas are not that difficult to grasp, overall, I found it very hard to follow them through the text. Therefore, the writing needs considerable work. I only state few of the points hoping that everything gets rectified through a thorough revision by the authors.*

*• Line 162: Definition of sheltered proportion missing.*

We have added the definition, thank you.(New lines 164-165)

*• Line 168: Interpretation missing – What is meant by weak effect of time? Can it be written more simply? No figure is referred to highlight this effect.*

By weak effect, we mean that although time has an influence on the total sheltered population (slope = 2.9x10^-5^, t =3.36, P < 0.001), this effect is small relative to the 22-hour test duration. The sheltered proportion increases with a slope of 2.9x10^-5^ minute^-1^. This means that there is only an increase of 0. 38 individuals between the 30th minute and the end of the experiment (1320 minutes, 22 hours). We have clarified this in the new version (new lines 172-174). Please also see the new figure highlighting this effect (new supplementary figure S3).

*• Line 191: Definition and unit of cumulative occupation?*

Is the sum of the observed presences of an individual (or individuals of a group) in a number of time steps. This cumulative is calculated for each shelter or for both. “occupation” was replaced by presence.

*• Line 192: Claim of difference between group choice and individual is unclear until the order parameter is defined clearly at the start.*

In such experiments (binary choices), when responses to different shelters are statistically equal, there is no discrimination. The quantification of these responses can be measured by numerous variables : number of entries, staying times and even the number of individuals present at each time on each of the sites and/or their cumulative presences, these last being used in our work. See section Shelter colour influences in the manuscript..

*• Line 195 - 198: Reference missing.*

Added, thank you.

*• Line 198: What thresholds are we talking about here? The authors cannot leave it for the readers to figure out themselves.*

You are right this idea was under-developed, please see the new version (lines 203-205).

*• In so many place use of comma is entirely missing. Just for an example, in line 205, the sentence should be – Indeed, if the quality of ... decreases, the decision ... (Note the use of comma).*

Thank you, please see new main text. We have improved the paper, taking into account your remarks.

*• Line 218: Isn’t the interpretation simple: that the groups are unable to respond quickly or react to environ- mental changes?*

Yes, we agree with your remark concerning this observation. But the question addressed: what are the mechanisms (interactions) involved in this difference, between isolated individuals and groups, and leading to stable or transitory bimodalities? see also below concerning your remark of line 227.

*• Fig 6 is referred before figure 5 in the main text. Hence, change their numbering order.*

You are right, please see new figures orders.

*• Line 227: The plot seems unimodal to me even after 660 minutes.*

You are right. In figure 6A it seems that bimodality emerges later in time (around 750 minutes). However, our statement is based on the Cramer-von Mises test. Please see also the supplementary the new figure S6, where the distribution of sheltered individuals between the shelter is shown for every time-step.

*• Definition of consensual fiedelity not clear*

Thank you, we clarified this. Please see the new lines 246-249.

*• Supplementary text line 121: isn’t it attraction of unsheltered to sheltered?*

The light intensity outside is such that the aggregation is inhibited. In our experiments, no aggregation was observed outside the shelters. In fact, in 80% of the observations, less than two individuals are simultaneously outside. therefore, by simplicity, we do not consider a social feedback influencing individuals to stay outside the shelters.

*• Supplementary text line 134: It should be mentioned that the solution is for a steady state by putting derivatives equal to zero.*

Done (see new lines 116 and 141)

*• Supplementary text line 206: Typos in equation.*

Done, see figure legend S10.
