# Supplementary material for: Emergence and retention of a collective memory in cockroaches
Source: PLoS One. 2023 Jul 6;18(7):e0287845. doi: 10.1371/journal.pone.0287845 (PMC10325095; doi:10.1371/journal.pone.0287845)
Supplement: Attachment — Submitted filename: Reviewer comments.docx [file pone.0287845.s013.docx]

Dear Editor,

We thank you and the reviewer for your constructive comments that have been addressed in the following point-by-point response and in the revised manuscript.

We hope that our manuscript “Emergence and retention of a collective memory in cockroaches” is now suitable for publication in PlosOne.

**Editor comments**

**Please review your reference list to ensure that it is complete and correct. If you have cited papers that have been retracted, please include the rationale for doing so in the manuscript text, or remove these references and replace them with relevant current references. Any changes to the reference list should be mentioned in the rebuttal letter that accompanies your revised manuscript. If you need to cite a retracted article, indicate the article’s retracted status in the References list and also include a citation and full reference for the retraction notice.**

We verified all cited references for formatting and possible retracted papers, for the latter non were found.

**Reviewer comments**

**Reviewer's Responses to Questions**

**Comments to the Author**

**1. If the authors have adequately addressed your comments raised in a previous round of review and you feel that this manuscript is now acceptable for publication, you may indicate that here to bypass the “Comments to the Author” section, enter your conflict of interest statement in the “Confidential to Editor” section, and submit your "Accept" recommendation.**

**Reviewer #1: All comments have been addressed**

**2. Is the manuscript technically sound, and do the data support the conclusions?**

**The manuscript must describe a technically sound piece of scientific research with data that supports the conclusions. Experiments must have been conducted rigorously, with appropriate controls, replication, and sample sizes. The conclusions must be drawn appropriately based on the data presented.**

**Reviewer #1: Yes**

**3. Has the statistical analysis been performed appropriately and rigorously?**

**Reviewer #1: I Don't Know**

**4. Have the authors made all data underlying the findings in their manuscript fully available?**

**The PLOS Data policy requires authors to make all data underlying the findings described in their manuscript fully available without restriction, with rare exception (please refer to the Data Availability Statement in the manuscript PDF file). The data should be provided as part of the manuscript or its supporting information, or deposited to a public repository. For example, in addition to summary statistics, the data points behind means, medians and variance measures should be available. If there are restrictions on publicly sharing data—e.g. participant privacy or use of data from a third party—those must be specified.**

**Reviewer #1: No**

Please find DOI for the repository of the experimental data available: <https://doi.org/10.6084/m9.figshare.21130477>. See also line 363 of the Revised manuscript.

**5. Is the manuscript presented in an intelligible fashion and written in standard English?**

**PLOS ONE does not copyedit accepted manuscripts, so the language in submitted articles must be clear, correct, and unambiguous. Any typographical or grammatical errors should be corrected at revision, so please note any specific errors here.**

**Reviewer #1: Yes**

**6. Review Comments to the Author**

**Please use the space provided to explain your answers to the questions above. You may also include additional comments for the author, including concerns about dual publication, research ethics, or publication ethics. (Please upload your review as an attachment if it exceeds 20,000 characters)**

**Reviewer #1: I am satisfied with the author's response and changes made to the manuscipt and the supplemental material. However, minor grammatical errors and typos still remain. I would lke to suggest that the captions of Table 1 and 2 in the main text can be expanded that will help the readers to understand the technical analysis performed there. Finally, I could hardly understand Figure 6 of the main text due to its poor quality. Apart from these concerns, tha manuscript seems good for a publication. The conclusions about collective memory inferred through intelligent experiments and a rigorous mathematical model are very interesting and should be welcomed by the community of researchers in this area.**

We thank you for your comments, please see the new captions of table 1 and 2 in the main text (lines 254-262 and 283-286 respectively) as well as the new uploaded figure 6.

**7. PLOS authors have the option to publish the peer review history of their article (what does this mean?). If published, this will include your full peer review and any attached files.**

**If you choose “no”, your identity will remain anonymous but your review may still be made public.**

**Do you want your identity to be public for this peer review? For information about this choice, including consent withdrawal, please see our Privacy Policy.**

**Reviewer #1: No**
